# Guest Editors' Introduction

**DOI:** 10.1186/1471-2105-13-S10-S1

**Published:** 2012-06-25

**Authors:** Jianer Chen, Ion Măndoiu, Raj Sunderraman, Jianxin Wang, Alexander Zelikovsky

**Affiliations:** 1 Department of Computer Science, Texas A&M University, College Station, Texas 77843, USA; 2 Department of Computer Science & Engineering, University of Connecticut, Storrs, CT, 06269, USA; 3 Department of Computer Science, Georgia State University, Atlanta, GA 30303, USA; 4 School of Information Science and Engineering, Central South University, Changsha, 410083, P.R. China

## 

This Supplement includes a selection of papers presented at the 7th International Symposium on Bioinformatics Research and Application (ISBRA), which was held on May 27-29, 2011 at Central South University in Changsha, China. The technical program of the symposium included 36 extended abstracts presented orally and published in volume 6674 of Springer Verlag's Lecture Notes in Bioinformatics series. Additionally, the program included 38 short abstracts presented either orally or as posters. Authors of both extended and short abstracts presented at the symposium were invited to submit full versions of their work to this Supplement. Following a rigorous review process, 19 of the 40 full papers submitted were selected for publication.

Selected papers cover a broad range of bioinformatics topics, ranging from algorithms for structural biology to phylogenetics and biological networks. The first two papers of the Supplement address two important problems in structural biology. Improved methods for predicting protein-protein and protein-DNA binding and identification of binding sites are critical components of rational drug design and functional annotation pipelines. The paper by Guo and Wang proposes an efficient algorithm for finding similar binding sites on the protein surfaces based on sequence alignment, protein surface detection, and 3D structure comparison. Validation experiments show significantly improved average recall and precision values compared with existing approaches.

Szabóová et al. propose methods for predicting protein-DNA binding propensity from spatial structure information without the use of evolutionary information. Such methods are particularly useful for optimizing DNA-binding of engineered proteins, for which evolutionary information is not available. Unlike previous approaches that rely on ad-hoc sets of physicochemical features, Szabóová et al. use ball histograms to capture the joint probabilities that amino acids complying with automatically selected properties occur within a certain distance of each other.

Rapid advances in high-throughput technologies are leading to ever increasing amounts of biological data. Four of the Supplement papers describe novel high performance computing methods needed to analize such massive datasets. Developing algorithms for specialized computing hardware such as GPUs (Graphic Processing Units) is increasingly being used by bioinformatics researches to solve large-scale problems, such as whole-genome sequence alignment, analysis of gene expression, and so on. Such a GPU-based high-performance method for 3D reconstruction in electron tomography (ET) is proposed in this supplement by Wang et al. They perform iterative reconstruction of ET on multi-GPUs via a multi-level parallel strategy combined with an asynchronous communication scheme and a blob-ELLR data structure. Another common way to achieve scalability is to improve the time-complexity of analysis algorithms, and next two papers of the Supplement adopt this approach. Eblen et. al present several improvements to the basic backtracking algorithm for finding maximal cliques in large graphs based on the concept of essential vertices. They observe that many biological data sets such as transcriptomic graphs derived from Gene Expression Omnubus (GEO) database, contain vertices belonging to every maximal clique. Experimental results provide significant speed-up in finding maximal cliques.

Skums et al. present two new error correction algorithms, KEC and ET, that are optimized for next-generation sequencing of viral amplicons. Compared to a previously published clustering algorithm SHORAH, the new algorithms show similar accuracy in finding true haplotypes, but are significantly more efficient in removing false haplotypes and estimating the frequency of true haplotypes.

In addition to scalability, accuracy is also of critical importance. For well studied problems such as clustering of genome-wide expression data there are numerous available algorithms, and selecting the most appropriate one can be a daunting task. Jay et al. provide a systematic comparison of 14 commonly used clustering algorithms, including traditional *k*-means and hierarchical clustering algorithms, several graph based approaches (both heuristic and clique-based), machine learning approaches such as self-organizing maps, and clustering algorithms specifically design for gene expression data such as QT Clust. Validation of obtained clusters against known gene classifications suggests that graph-based methods consistently outperform the other methods.

Traditionally, computational problems related to evolution are very well represented at ISBRA. In this supplement six papers are devoted to computational issues in phylogenetics. By using bioinformatics and systems biology approaches, Zhan Zhou et al. studied the genes' function and systems evolution in five species of Streptomyces. Their results provide evidence of genome plasticity and rapid evolution within the five species of Streptomyces. They show that genome diversification within this genus can be in part explained by frequent gene duplication or lateral gene transfer events since multiple single gene expansion and chromosome block expansion are observed. The paper provides a catalog of genome components and their potential functionality.

Zheng and Sankoff adopted and improved techniques for extracting large and paralogy-free multiple orthologies from conflated pairwise synteny data and approaches for ancestral gene order reconstruction in a given phylogeny, and evaluated the hypothesis that the order Malpighiales belongs to the malvids rather than as traditionally assigned to the fabids. Their results show that gene orders of ancestral eudicot species can be reconstructed in an efficient, parsimonious, and consistent way, despite paralogies due to whole genome duplication and other processes.

In the paper titled "Guided evolution of in silico microbial populations in complex environments accelerates evolutionary rates through a step-wise adaptation" Mozhayskiy and Tagkopoulos provide simulation evidence to their hypothesis that the rate of evolution of microbial populations can be both accelerated and decelerated by exposing the populations to a series of environments of varying complexity. They have developed a state of the art scalable microbial simulator that runs on a high performance computing environment. The work has implications in many areas of biological research including synthetic biology and bioengineering research.

In the next paper, Chaudhary et. al present innovative algorithms to solve the gene-tree species tree reconciliation problem. Traditional gene tree parsimony approaches can produce biased results with errors in gene trees. The algorithms presented in the paper search local subtree prune and regraft (SPR) or tree bisection and reconnection (TBR) neighborhoods of a given gene tree to identify a topology that imply fewest duplications, losses, or deep coalescence events. The algorithms also provide a fast error correction protocol for gene trees, in which small rearrangements in the topology are allowed to improve reconciliation costs. Finally, the paper introduces a simple protocol to use the gene rearrangement algorithm to improve gene tree parsimony phylogenetic analyses.

In the paper by Lin, Burleigh, and Eulenstein it is shown that the deep coalescence consensus tree problem satisfies the highly desirable Pareto property for clusters, in the sense that in all instances, a cluster present in all of the input gene trees will also be found in every optimal solution. They propose a divide and conquer algorithm for the deep coalescence problem based on the Pareto property. Analysis of simulated and empirical data sets demonstrates that the new algorithm greatly improves the computational speed of heuristics that do not consider the Pareto property.

In the paper titled "Horizontal gene transfer dynamics and distribution of fitness effects during microbial evolution" Mozhayskiy and Tagkopoulos introduce a new simulation framework for studying the effects of horizontal gene transfer on microbial evolution. The approach presented in the paper is able to study the effect of horizontal gene transfer on the evolution of complex traits and biological networks, which is not possible with previous approaches. Biological networks and gene expression dynamics are elegantly incorporated into the simulation providing great insight into actual cellular and evolutionary processes. In order to reconcile un-rooted and erroneous gene trees by simultaneously rooting and error-correcting them, Pawel Gorecki et al. develop an efficient algorithm by searching neighborhoods of the given un-rooted tree. They reduce the time complexity of this problem to *O*(*lk *+ *max*(*m*, *n*)), where *n *and *m *are the sizes of the gene tree and species tree respectively, *l *is the number of weak edges in the gene tree and *k *is a small value which indicates the size of the neighborhoods.

The most important problem in the post-genomic era is relating genes and their functions. Gene expression data is widely used in the functional analysis of genes. Recently, considering the complicated relationship among genes, researchers proposed various set-level methods to identify prior defined sets of genes that are differentially expressed across sample classes. Matej Holec et al. present an evaluation of these methods in the classification of gene expression samples.

In the paper titled "Exploring Biological Interaction Networks with Tailored Weighted Quasi-Bicliques", Chang et. al present novel algorithms to identify functional modules in biological networks that are represented as bipartite graphs. They introduce the concept of edge-weighted quasi-bicliques that capture biological interaction levels and despite proving that in general the problems are NP-Hard, they present Integer Programming formulations that provide fast solution for moderately sized networks.

In the next paper, Hu and Jiang present a novel approach to disease classification using microarray gene expression data. They introduce a gene module or cluster based extension to the standard linear discriminant analysis statistical technique. The rationale behind their approach is that genes act in networks and modules identified from these networks can be used as features in constructing a classifier. The new method (MLDA) introduced is shown to perform better than other existing methods on three real data sets: colon, prostate, and lung cancer microarray data.

Jancura et al. proposed a computational methodology for detecting the orthology signal present in a protein-protein interaction (PPI) network, and developed a filtering technique for extracting orthology-driven clusters with unique functionalities. They applied their methodology to the Yeast PPI networks and the results show that evolutionary information at a functional complex level can be retrieved from the structure of the network.

Jieyue He and her colleagues studied the gene functions from network perspective. To accurately identify protein functional modules in protein interaction networks, they proposed a core-attachment based greedy search method (GSM-CA). Furthermore, to reduce the computational cost of traditional hierarchical methods, they also developed an efficient fast clustering based greedy search method (GSM-FC).

Jiang and Wu extend several methods previously used to predict protein function to predict subcellular protein localization from the PPI network, based on the observation that proteins that interact *in vivo *are often co-localized in the same cellular compartment. The authors present the results of a comparative study of the proposed methods conducted on a large yeast dataset, demonstrating good prediction accuracy.

In summary, this Supplement to BMC Bioinformatics includes 19 articles presenting recent advances in bioinformatics research and applications. We hope you find them as useful and as interesting as we do.

## Competing interests

The authors declare that they have no competing interests.

